# Fetal Programming and Its Effects on Meat Quality of Nellore Bulls

**DOI:** 10.3390/vetsci10120672

**Published:** 2023-11-24

**Authors:** Arícia Christofaro Fernandes, Mariane Beline, Guilherme Henrique Gebim Polizel, Roberta Cavalcante Cracco, Evandro Fernando Ferreira Dias, Édison Furlan, Saulo da Luz e Silva, Miguel Henrique de Almeida Santana

**Affiliations:** Department of Animal Science, College of Animal Science and Food Engineering—USP, Av. Duque de Caxias Norte, 225, Pirassununga 13635-900, SP, Brazil; mbeline@vt.edu (M.B.); guilherme.polizel@usp.br (G.H.G.P.); edisonfurlan@usp.br (É.F.);

**Keywords:** adipose, beef cattle, maternal nutrition, muscle development, prenatal supplementation

## Abstract

**Simple Summary:**

Population growth, which generates a worldwide concern regarding the supply of food to meet this demand, is expected in the coming years. In Brazil, beef cattle farming remains significant, but the sector aims to increase its share of the supply of beef to the global market. Therefore, the adoption of strategies to obtain more productive and higher quality animals becomes essential. Nutrition is one of the main pillars of animal performance and can directly contribute to this objective, even more so when it happens even before the animal is born, via maternal nutrition. The importance of this lies in taking advantage of windows of fetal development linked to the formation of muscle and fat. For this, we used analyses of carcass yield and meat quality to assess how the diet of the mother at different stages of pregnancy affects the offspring. And we found that it was not possible to observe changes in the meat, but there is evidence of alterations in the tissues that make up the meat. Much is linked to the level of supplementation that the females received and not exclusively to the gestation period. We suggest further studies to clarify the factors that directly influence meat.

**Abstract:**

This work aimed to evaluate the effects of prenatal nutritional stimulation at different pregnancy stages on carcass traits and meat quality in bovine progeny. For this purpose, 63 Nellore bulls, born from cows submitted to three nutritional plans, were used: not programmed (NP), which did not receive protein supplementation; partially programmed (PP), which had protein-energy supplementation (0.3% of mean body weight of each batch) only in the final third of pregnancy; and full programming (FP), which received supplementation (0.3% of mean body weight of each batch) throughout pregnancy. The averages of parameters were submitted to the ANOVA, and the supplementation periods, which were different when *p* value < 0.05, were compared. Carcass weights and rib eye area (REA) did not differ between treatments (*p* > 0.05), but subcutaneous fat thickness (SFT) showed a tendency (*p* = 0.08) between groups. For lipids and marbling, no differences were found (*p* > 0.05). In the analyses of maturation time and shelf life, no difference was observed between treatments. However, there was a tendency between treatments at 14 days of maturation time for cooking loss (CL) (*p* = 0.08). Treatments did not affect shear force in the progenies (*p* > 0.05). Fetal programming had no effect on the meat quality of Nellore bulls.

## 1. Introduction

The demand for beef cattle yield and quality has increased worldwide [1]; however, the beef production cycle faces several difficulties in the environment and nutrition, which directly affect the growth and performance of meat-producing animals [2]. In beef cattle, the carcass that originates the meat cuts is basically composed of three main tissues, which ensure meat yield and quality: skeletal muscle, adipose tissue, and connective tissue. The formation of these tissues begins during pregnancy and occurs from the processes of myogenesis, adipogenesis, and fibrogenesis [3].

These processes involve the differentiation of multipotent cells into muscle fibers, mature adipocytes, and fibroblasts [4]. Therefore, animals can develop and grow via two stages, hyperplasia (increase in cell number) and hypertrophy (increase in cell volume), which are the main aspects responsible for efficiency, quantity, and beef quality at the end of the production cycle [5].

Regarding muscle tissue, fiber hyperplasia occurs exclusively in the fetal period, while only hypertrophy occurs in postnatal life, which can extend until the end of puberty [3]. As for adipose and connective tissues, hyperplasia begins in the middle of pregnancy and is not limited to the fetal phase. Hyperplasia occurs until the neonatal phase for visceral fat and until weaning for subcutaneous and intermuscular fat. And, finally, intramuscular fat increases its cell count until about 250 days of age [6,7]. After these periods, the organism only provides for fat accumulation in the respective deposits, and fibroblasts accompany the deposition of adipose tissues.

Myogenesis can be divided into primary myogenesis, which occurs in the first two months of pregnancy and is the fiber mold for secondary myogenesis, which is formed from the 2nd to the 7th month of pregnancy [8,9,10,11]. The availability of nutrients that the fetus uses to develop and grow these tissues during pregnancy come exclusively from the mother; therefore, special care is required with the nutrition of the pregnant cow [12]. The major concern is that maternal nutrition may not support the complete development of these tissues due to the low availability of food or nutrients consumed/supplied during the months of pregnancy [13]. This requires directing the ingested energy to tissues of greater preference, such as organs and vital systems for survival, thus affecting tissues with less priority, such as skeletal muscle [14,15], reducing meat production and quality.

This nutritional deficiency faced by pregnant cows is largely due to the type of farming system most used for this category in the country, which involves a continuous pasture system over large areas [16]. This type of system poses a challenge to livestock farming, since forage production is susceptible to climate conditions. Annual climate changes affect the food availability for animals based on the forage production seasonality, representing a hurdle in periods of rain shortage and less luminosity [17]. In Brazil, dams are predominantly of the Nellore breed, animals with the genetics developed for tropical conditions; however, due to the extensive system, little or no technology is used in the nutrition of these females [18]. This becomes a problem, as the reproductive calendar of these cows does not follow the availability of forage in the field. The final third of gestation until the beginning of lactation, which are the periods of greatest demand, occur precisely during the dry period, with low quantities and quality of forage available [19]. Therefore, one of the strategies to minimize the impacts of this system is the use of supplementation for these mothers during pregnancy, ensuring the supply of nutrients for fetal development.

The challenge faced by the cow during pregnancy, which causes alterations in the progeny, has become known as fetal programming. The term fetal programming, or developmental programming, refers to a positive or negative stimulus during pregnancy, promoting a differentiated uterine environment for the growing fetus and altering the developmental trajectory in postnatal life [20].

Thus, to meet the demand of beef consumers, while tackling the nutritional challenges faced by cattle dams in certain periods of the year, the present study investigated the effects of nutritional stimulus during the gestation of Nellore cows on the carcass features and meat quality of male progeny.

## 2. Material and Methods

### 2.1. Ethics Statement

All procedures were approved by the Committee on Ethics in the Use of Animals, for the Faculty of Animal Science and Food Engineering at the University of São Paulo—FZEA/USP (CEUA/FZEA), under protocol No. 1843241117. The care and use of the animals were performed according to the rules of the National Council for the Control of Animal Experimentation (CONCEA).

### 2.2. Experimental Design

For this study, 126 Nellore cows were used initially; all were multiparous and underwent a fixed-time artificial insemination protocol with semen from four Nellore bulls, being the predominant breed in the country. After confirmation of pregnancy, the cows were assigned to the treatments according to age (3–8 years), body weight (BW), and body condition score, measured at the time of insemination, to keep the batches as homogeneous as possible, as described by Cracco et al. [21]. The cows remained on pasture of the species *Brachiaria brizantha* cv. Marandu throughout the experiment, in a rotational grazing system, with each treatment rotated in three different areas. All cows received a mineral supplement (0.03% average body weight of each batch) and water was offered ad libitum during the entire pregnancy. The experiment was performed in a completely randomized design in which each dam was considered an experimental unit. The 126 cows were divided into three treatments, namely nutritional plans, which provided or did not provide nutrients to the pregnant cow at different times of pregnancy. The treatments comprised: not programmed (NP) or control, which did not receive the energy protein supplement, partially programmed (PP), which consumed the supplement only in the final third of pregnancy, and full programming (FP), which received the supplement throughout pregnancy. Both PP and FP groups received a daily supplement corresponding to 0.3% of the average body weight of the cows until calving, in accordance with the National Research Council’s (NRC, 2000) [22] nutritional, maintenance, and gain recommendations for cows during mid- to late pregnancy. Table 1 presents the ingredients and nutrients in the supplement offered to pregnant cows, while Table 2 shows the nutrients in pastures. These tables are also present in the methodology of Schalch Junior et al. [23]. After calving, all cows were grouped into a single batch under the same environmental conditions with the same nutritional treatment (mineral supplementation); therefore, the nutritional stimulus was exclusively prenatal.

The dams were assessed regarding their BW, body condition score, and rump fat thickness during pregnancy, and the results showed effects of the supplement on the performance of these females during the experiment. FP cows showed greater BW and fat thickness during the final third of pregnancy and pre-partum, compared to NP and PP. However, the groups displayed equal values for these characteristics in the initial period of pregnancy and post-partum. The body condition score was different between the NP and FP groups pre-partum, while no difference was found at the beginning of pregnancy, in accordance with Cracco et al. [24].

Male offspring of the dams mentioned above, more specifically, 63 non-castrated males of the Nellore breed with an average age of 22 months, were used to evaluate meat quality traits. The males were reared on pasture for 8 months with their mothers and were subsequently submitted to an 11-month rearing period on pasture and supplementation in a trough, followed by an average of 106 days of finishing in feedlot. From birth to slaughter, all animals were submitted to the same environmental and nutritional conditions (sanitary management, experimental collection, and feeding). Male breeding and rearing phases were developed under the same pasture system (rotated in *Brachiaria brizantha* cv. Marandu). In the rearing period, the animals received an energy supplement of 0.3% of the average weight of the batch during the 6 months in the dry season (winter), (TDN = 67.55%; CP = 24.78%; NDF = 11.24%; fat = 2.61%; 0.3% of BW), and protein supplementation in the 6 months following, which corresponds with the wet season (summer), (TDN = 53.15%; CP = 30.03%; NDF = 9.14%; fat = 1.65%; 0.1% of BW) with the offer of 0.1% of the average batch weight, until the animals entered the feedlot, according to Polizel et al. [25].

During the feedlot period, three different diets were formulated, but the same diet was offered to all animals. At the end of 98 days in the feedlot, the animals underwent the last collection of material and data. Slaughter occurred every 7 days and, on each day, 21 animals were slaughtered, seven of each treatment. The criterion to select the order of the slaughter groups was the subcutaneous fat thickness (SFT) obtained in the last carcass ultrasound (pre-slaughter), performed by a technician certified by the Ultrasound Guidelines Council (UGC), according to the methodology contained in the Beef Improvement Federation (BIF), between the 12th and 13th ribs. For the ultrasound evaluation, the Aloka SSD-500 ultrasound equipped with a 17.2 cm linear transducer at a frequency of 3.5 MHz (Aloka Co., Ltd., Wallingford, CT, USA) was used. Vegetable oil was used as a coupling to optimize the contact of the transducer with the animal skin [26]. From this, an image file was generated that underwent Lince^®^ software analysis 6.0 (M&S Agricultural Consulting Ltd., Pirassununga, SP, Brazil) to read and generate the measurements. Based on these data, slaughter began with the group of animals with the highest SFT in each treatment, followed by the group characterized by average SFT, and ending with the group of animals with the lowest SFT for the third week of slaughter.

From birth until slaughter, males were evaluated for body weight, rib eye area, and subcutaneous and rump fat thickness. However, no difference was observed between treatments for carcass evaluations throughout this period. The same was true for BW, where performance was similar between the groups, with the exception of the weaning period, in which PP and FP had greater BW compared to the NP group (control), but this difference was not seen in any other time, per Cracco et al. [24].

### 2.3. Slaughter and Carcass Traits

The animals were slaughtered after a 12 h fast. At the end of the process, the carcasses were weighed individually (hot carcass weight—HCW), washed, and sent to the refrigeration chamber (0–2 °C) for 24 h for the rigor mortis process. On the following day, the carcasses were removed from refrigeration and weighed again in the same entry order to observe cooling loss (cold carcass weight—CCW). Subsequently, the sirloin steak (*Longissimus thoracis* et *lumborum*) was removed from the left half of the carcasses for the quality analyses.

In the *Longissimus lumborum*, the rib eye area (REA) and SFT were recorded in the transverse portion of the cut at the level of the penultimate rib (12th) in the cranial direction, with an image from a digital camera attached to a support to fix the distance between the equipment and the cut surface, standardizing the analysis. The REA and SFT were evaluated individually in digital images via manual delimitation of the regions of interest using the Lince^®^ software (M&S Agricultural Consulting Ltd., Pirassununga, SP, Brazil). Then, the cut was deboned and cut into 2.5 cm steaks as samples.

### 2.4. Meat Quality Analyses

#### 2.4.1. Marbling Score

The marbling score was estimated via visual assessment using a single trained evaluator comparing six different degrees (1 = mild; 2 = small; 3 = modest; 4 = moderate; 5 = slightly abundant; 6 = moderately abundant). Within each degree, there were four readings (0, 25, 50, and 75), according to the abundance of intramuscular fat in the samples, where 0 showed the lowest abundance and 75 indicated the highest presence of fat, following the USDA grading system. The score was converted into a 24-point numeric scale, being the combination of one degree with one reading (6 grades × 4 readings) to enable the statistical analysis of the data generated.

#### 2.4.2. Total Intramuscular Lipid

Three grams of the sirloin sample were weighed and homogenized in a solution of chloroform, methanol, and distilled water (2:1:0.8), using a processor (Mixer Walita Model RI1364 with microprocessor, Phillips do Brazil LTDA, Varginha, Brazil). Then, 1.5% NaCl was added to isolate and determine the lipids via gravimetry, according to the methodology proposed by Bligh and Dyer [27]. Chloroform allows separating the homogenate into two phases, chloroform and lipids, which was subsequently oven dried to evaporate the reagent, leaving only the lipid part. To determine the values, all containers were weighed individually before and after applying the methodology and always kept in desiccators to reduce interference from the environment.

#### 2.4.3. Shelf Life

For the shelf life test, a sample of *Longissimus lumborum* (1.5 cm thick) was collected and divided into three similar parts to compose the subsamples (time 0, 3, and 5 days). Subsample 0 was analyzed still in the processing room at deboning (24 h after slaughter). This test comprises the color and pH readings, according to the method proposed by Vatansever et al. [28]. Samples 3 and 5 were placed on polystyrene trays containing absorbent paper and wrapped in oxygen-permeable film under simulated retail exposure conditions (4 °C and 1000 lux lighting) for further analysis in the laboratory. Before color and pH readings, the subsamples were exposed to the environment (refrigerated display) without the polystyrene cover for 30 min at 4–6 °C to allow the reaction of myoglobin with O_2_ (blooming). Next, an evaluation of the color was carried out at an approximate temperature of 6–8 °C using a portable spectrophotometer, model CM2500d (Konica Minolta Brazil, São Paulo, Brazil) with standard illuminant A, observation angle of 10°, and shutter aperture of 30 mm. Final values of L* (brightness), a* (red intensity), and b* (yellow intensity) of each sample were obtained using the average of three observations at different points of the sample, avoiding points that could interfere with the reading. The pH was measured using a digital meter (Hanna Instruments model HI99163, São Paulo, Brazil), inserted in a random point of the sample with the complete insertion of the crystal, according to the CIELab system [29], for the three measurement times (0, 3, and 5 days).

### 2.5. Maturation, Cooking Loss, and Warner–Bratzler Shear Force

After collecting the shelf life samples and maintaining the craniocaudal direction, three more samples (2.5 cm thick steaks) were collected from the sirloin steak, which were individually identified and vacuum packed to be matured for 0, 7, and 14 days. After the maturation times in a refrigerated chamber (2–5 °C), the samples were removed from the vacuum packages and exposed to the environment for 30 min to allow for *blooming*. Then, an objective evaluation of color and pH was carried out according to the CIELab system [29]. This methodology was applied for the three maturation times 0, 7, and 14 days.

After the color and pH analysis, the samples were weighed on a precision scale and baked at 170 °C in an industrial electric oven (Model F130/L—Golden Arrow Electric Ovens Industry and Commerce Ltd., São Paulo, Brazil) equipped with a thermostat. The internal temperature of the steaks was monitored using individual thermometers (Inkbird Tech C.L.—Model IBT-6XS, Smart BBQ thermometer, Shenzhen, China). Upon reaching an internal temperature of 40 °C, the samples were turned over and remained in the oven until they reached an internal temperature of 71 °C, as recommended by the American Meat Science Association [30]. The samples remained at room temperature until cooling at ±25 °C, when they were weighed again to determine cooking loss (CL).

After, the samples were wrapped in plastic film and refrigerated (4–6 °C) for 24 h to start the Warner–Bratzler shear force (WBSF) methodology. Afterwards, six cylinders (1.27 cm diameter) were removed from each sample, parallel to the fibers, to determine the WBSF using the TMS-PRO texture analyzer equipment (Food Technology Corporation, Sterling, VA, USA) coupled with a Warner–Bratzler shear device, with a fixed speed of 200 mm/min [30]. The WBSF of each sample was considered as the average of the repetitions.

### 2.6. Statistical Analysis

The effect of the treatment on the variables studied was evaluated via the analysis of variance (ANOVA), considering the fixed effects of the nutritional treatment of cows (NP, PP, and FP) and the times of analysis, with the day of slaughter as a random effect. The analyses were conducted using the MIXED procedure of SAS software (SAS Institute Inc., Cary, NC, USA). Shelf life, maturation, cooking loss, and shear force were evaluated as repeated measures over time, and the interaction between treatment and time was included in the models as a random effect. The covariance dams were tested for each trait, and the best fit was used [31]. The means of the treatments were obtained using the LSMeans procedure, and, in case of differences between the treatments, the means were compared using the Tukey Test with a significance level of 5%, allowing for a tendency when 5% < *p* value < 10%.

## 3. Results

### 3.1. Carcass Traits

There were no significant differences between the treatments for animal carcass traits (rib eye area and subcutaneous fat thickness—SFT); nevertheless, SFT tended to be higher in the FP treatment (*p* = 0.08). Hot carcass weight and cold carcass weight were not affected by the prenatal nutritional stimuli of males evaluated after slaughter (*p* > 0.05) (Table 3).

### 3.2. Meat Quality

#### 3.2.1. Marbling Score and Total Lipids

The results obtained from the analyses of total lipids and marbling score showed no significant difference between the groups of the different prenatal nutritional strategies (*p* > 0.05) (Table 3), although the PP treatment was inferior in both analyses.

#### 3.2.2. pH and Color in Shelf Life

The pH and color of the shelf life analyses did not show significant differences between treatments (*p* > 0.05). However, color components L* (*p* = 0.10) and b* (*p* = 0.09) showed a tendency at 24 h after slaughter (D1). The pH, a*, and b*, when analyzed over time, showed no statistical difference (*p* > 0.05), although b* still showed a tendency in the periods when the steaks were analyzed (*p* = 0.07). However, the L* color component showed a significant difference between shelf life times (*p* < 0.05). No parameter analyzed in shelf life showed treatment x time interaction (*p* > 0.05) (Table 4).

#### 3.2.3. pH and Color at Maturation

With the exception of the a* color component, which showed a significant difference over time (*p* < 0.05), the other parameters of this analysis did not show statistical differences for treatments and maturation times (Table 5). Moreover, there was no interaction between the effects of animal treatments and the times of the methodology used (*p* > 0.05).

#### 3.2.4. Cooking Loss and Shear Force

For the CL analysis, there was a tendency (*p* = 0.08) for the treatments to differ at time D14, with the FP treatment showing lower cooking loss values. The same analysis did not show differences in time, but there was a significant interaction of treatment with the aging times of the steaks (*p* < 0.05) (Table 6). The WBSF evaluations did not show a significant difference between the groups tested. The same result was observed for time and for the time x treatment interaction (*p* > 0.05), showing no influence of fetal programming on the steak tenderness of the beef samples (Table 6).

## 4. Discussion

### 4.1. Carcass Weight and Rib Eye Area

Carcass weight and rib eye area (REA) are the indicative measurements of the animal meat yield and relate mainly to skeletal muscle, the main component of carcasses [32]. The muscle fibers that compose the skeletal muscles are formed only during pregnancy, from cell hyperplasia, which indicates the importance of cow nutrition on the fetus potential to develop this tissue, in addition to initiating hypertrophy, which increases muscle mass [3,33]. Some studies have analyzed the effects of the nutrition of pregnant cows on the carcass traits of calves, including BW and REA. The literature reports an antagonism of results, which show the influence of maternal intake during pregnancy on carcass weight and/or REA [34,35,36,37,38,39]. And studies, such as our study, found no effect of the nutritional stimulus of mothers on the offspring in relation to both traits [34,36,38,40,41,42,43,44,45]. Therefore, further studies are needed to understand the mechanism of action of different diets and/or nutrients, which stimulate different responses related to the development of skeletal muscle tissue. For instance, Marquez et al. [34] evaluated the profile of muscle fibers and reported a difference in the REA and the number of muscle fibers in programmed animals, without changing the size of fibers. In contrast, Polizel et al. [46] concluded that animals supplemented in the final third of pregnancy and throughout pregnancy had greater BW without modifying the REA or the number and area of muscle fibers in bovine rearing. This means that similar studies on fetal programming, using supplementation at certain periods of pregnancy, but with different ingredients and nutritional plans, motivated a priori greater hyperplasia, as in the case of Marquez et al. [34], and greater hypertrophy in the study by Polizel et al. [46]. These findings indicate the plurality of results that maternal nutrition can generate to the offspring.

The absence of significant differences in HCW and CCW shows that the animals also had similar drip loss, maintaining their postmortem weights statistically equal. Drip loss is directly linked to the pre-slaughter handling of the animals, carcass temperature when entering the cooling chamber, pH drop in rigor mortis, and the size and thickness of carcass superficial fat [47]. All of these parameters have an effect on the rigor mortis process and can denature proteins and alter the structures of fibers, promoting the shortening of the sarcomere, thus forcing the water outflow from the cells [48]. The decrease in weight due to tissue exudation and superficial evaporation also affects meat quality parameters; the same non-significant result for offspring hot carcass weight was found in sows that received diets with adequate or excessive energy density during gestation, according to Lugarà et al. [49]. Similarly, Meale et al. [50] did not observe the influence of the maternal diet at the beginning and middle of pregnancy on the weight of the hot carcass, in animals with different genetic potential for residual consumption.

### 4.2. Subcutaneous Fat Thickness

In the partition of nutrients, subcutaneous fat is the second adipose tissue to be developed in the fetal stage, starting around the middle third of gestation, and its adipocytes continue to multiply until the beginning of weaning [7]. After this period, the animal only increases the size of these cells, especially in the termination phase. Subcutaneous fat is very important in the quality meat process, as a minimum thickness is necessary for a protective layer in the carcass cooling process, acting as a thermal insulator and preventing the cold shortening of fibers [51]. The minimum thickness required in industries to prevent cold shortening is 3 mm [52,53]. Although the results for SFT in the present study did not show significant differences between treatments, there was a tendency for this trait (*p* = 0.08), and all treatments had more than double the minimum thickness required. Similar to our results, which presented from 7.81 to 8.89 mm, Long et al. [54] found an average thickness of 10 mm of subcutaneous fat, which was not significant between dams fed only 55% and 100% of the requirement indicated in the National Research Council, Washington, DC, USA (NRC, 1996), thus also did not change the SFT of calves. In addition, malnutrition in the initial third of pregnancy did not promote changes in the degree of carcass adiposity in male cattle [55].

Moreover, there was no difference in the SFT of the offspring in the maternal protein restriction during the middle and end of gestation [41], and the supplementation of protein sources at the end of pregnancy was not enough to change the SFT of the progeny [43,45]. In contrast, Underwood et al. [39] observed that dam nutrition, when improved from mid- to late pregnancy, resulted in a greater fat thickness of the 12th rib of bulls. In addition, higher protein intake before parturition increased the coverage adipose of offspring [42], and when this consumption was above the nutritional requirement of the pregnant cow in the prepartum period, it also increased the deposition of subcutaneous fat [38]. These discordant results may be related not only to the period, but also to the quality and quantity of the nutrients tested in those studies. In our study, the absence of statistical differences in the traits that indicate adiposity may suggest that the nutritional restriction of the NP treatment or the stimuli of the PP and FP treatments were not enough to cause changes in the adipogenesis of the tested males.

The nutritional strategies adopted in our study during pregnancy were not sufficient to change the percentage of total lipids and the marbling score of bulls. Similar results were reported with no effects of maternal nutrition during early pregnancy [54], in the middle of pregnancy [37], or even in the middle to the end of pregnancy on the intramuscular fat deposition of the progeny, due to different supplement levels [38,39,56,57]. The presence of intramuscular lipids and the formation of marbling, as well as other body fats (adipogenesis), are basically the consequences of two processes known as hyperplasia, which concerns the formation of fat cells (adipocytes), and hypertrophy, which is the increase in the volume of these cells [5]. The increase in intramuscular fat, which confers the presence of lipids in the meat and the different marbling degrees, is dependent not only on hypertrophy, but mainly on hyperplasia, which allow a greater concentration of cells to distend during the fattening period [7]. Adipogenesis begins during the middle third of gestation; however, multipotent cells, which originate adipocytes, have a priority in tissue differentiation at the time of development and animal growth. This means that there is the development of visceral fat first, followed by the deposition of subcutaneous adipose tissue intermuscular and, finally, intramuscular fat [58]. Despite the hypothesis that marbling is more efficient in the intrauterine phase [59], due to the number of multipotent cells inversely proportional as the age of the animal increases [6], other studies have shown that the neonatal period is also efficient in the formation of intramuscular fat, similar to the fetal period, because the priority of cell differentiation decreases in other adipose tissues, which can better direct nutrition toward marbling formation [59,60]. This idea of a period of intramuscular adipocyte formation without increasing overall adiposity is called “marbling window”, which comprises the interval from the final third of gestation to approximately 250 days of age [5,7]. Therefore, the animal has almost ¾ of its potential for intramuscular fat formation after birth, in which the nutrition from breastfeeding is possibly responsible for this deposition, corroborating with [61], who reported that super nutrition of mother mice during lactation promoted the development of adipose tissue in the offspring. The possibility of extra supplementation (creep feeding) could have a greater effect on promoting subcutaneous fat thickness, when compared to maternal supplementation only in the final period of pregnancy. Therefore, postnatal nutrition, until mid-weaning, is likely to have more impact than fetal programming on marbling formation and lipid presence, since the critical window of intramuscular fat adipogenesis is greater after partum [7,62]. This shows that the results obtained in this study reflect what was expected regarding the presence of marbling and lipids in the meat of programmed animals.

### 4.3. pH Level

The pH is a condition that can alter many other traits of meat quality, and its variation depends on factors such as pre-slaughter stress, fat finish, and carcass temperature, among others [63]. Although there were no significant differences in the pH levels between treatments or times, the raw values had a similar behavior for both analyses (shelf life and maturation), with the tendency to be higher in the NP treatment in relation to the other groups and smaller over time compared to PP and FP. For both analyses, the pH remained within the range of 5.4 to 5.8, considered adequate for the Nellore breed for a good shelf life of the meat [64]. Other studies have reported similar results [55,65]; however, Meale et al. [50] observed differences in the carcass pH, with higher levels for animals whose mothers received a greater nutritional contribution than the other group during the beginning and middle of pregnancy.

### 4.4. Meat Color

Color is one of the most important factors in meat quality, as it is directly related to the acceptance of the product by the consumer, and it is the first characteristic that the customer evaluates when choosing the meat on the display [66,67]. The meat with a brighter/brighter red color is preferred by most consumers [68] and its variations are basically determined by the myoglobin behavior [55]. The presence of myoglobin is related to the type of muscle fiber. Type I fibers are classified as slow contraction, using mainly fat as an energy source and with an aerobic metabolism rich in mitochondria and myoglobin. Type II fibers, on the other hand, are characterized by a high contraction speed, using glucose as an energy source, and poor in myoglobin, which gives the meat more pallor [69]. These energy sources in ruminants are obtained via the metabolism of short-chain fatty acids (acetate, propionate, and butyrate), which come from ruminal microbial fermentation from foods rich in carbohydrates and fats (roughage and concentrates). During pregnancy, the nutrients available for the development of the embryo and fetus are generated exclusively by the diet of the mother [3,70]; therefore, the formation of the muscle tissue of offspring comes from the type of nutrient ingested by the cow. Myogenesis forms type I and type II fibers and occurs only in the fetal phase and from the 2nd to the 7th month of gestation [6,33]. Thus, the use of food supplements, in addition to pasture, can direct the muscle construction with specific fibers. The groups that received supplementation with non-structural carbohydrates, either in the final third of or throughout the pregnancy, tended to have a greater amount of type II fibers and consequently less myoglobin, which gives a lighter and less reddish color in relation to males whose mothers only grazed, consuming only roughage. Our work corroborates this idea, as the results obtained from the reading of the color components L* and b* tended to show differences between the groups tested in the shelf life analyses. For the results over time, the color components L* (maturation) and a* (shelf life) showed a difference between days, and the component b* (shelf life) showed a tendency, results that are related to the myoglobin behavior from the presence of oxygen in the environment, expressing the altered color. Nevertheless, it is necessary to evaluate the fibers of these animals to elucidate the effect of cow nutrition on the type of fiber in the muscle. The same non-significant effect of color was also observed by other authors after dietary restriction [37,65,71,72].

### 4.5. Warner–Bratzler Shear Force

The average of the Warner–Bratzler shear force (WBSF) of the treatments, evaluated in the three times (D1, D7, and D14), ranged from 53.94 to 79.11 N; that is, regardless of the treatment and maturation period, all treatments produced, on average, progenies with steaks that could not be certified as tender, as WBSF values above 45 N are commonly observed in Zebu breeds [73]. Even without significant differences between treatments, the shear force was lower over time for all groups. This result was expected due to the action of proteolytic enzymes on the fiber structures, degrading them and increasing tenderness, after the maturation times [74,75,76,77]. Alvarenga et al. [65] found the same non-significant results for shear force in bulls that underwent protein restriction during their prenatal phase. In contrast, Ref. [39] found a lower WBSF in the offspring of mothers that consumed better pastures, in relation to the dams fed with native pasture. The importance of fetal programming based on maternal supplementation on meat tenderness is observed in the preparation the animals for a shorter production cycle, making these individuals reach slaughter time younger, with good muscle development, with the presence of marbling, and with a good finish of subcutaneous fat. These factors are decisive for meat with lower WBSF values, since young animals have less collagen and more thermo-soluble collagen compared to older animals [3,39,78]. In addition, nutrition during pregnancy, as already discussed, influences the deposition of subcutaneous and intramuscular fat, which directly affects meat tenderness [3].

### 4.6. Cooking Weight Loss

Cooking loss (CL) is the liquid content released in the process of heating the meat, which can alter the quality by reducing the nutritional value (the leaching of vitamins and minerals), altering texture (juiciness and flavor), and modifying meat composition (protein denaturation and connective tissue solubilization) [79,80,81]. Both CL and WBSF are influenced by the reduction in pH in the rigor mortis process, due to the alteration of enzymatic reactions that degrade muscle fiber structures, reducing the meat water retention capacity. In addition, the heat treatment can denature water-bound proteins, increasing the exudation rate. The maturation process also contributes to increased CL, as proteolysis continues over time [82]. The temperature and cooking time are determinant in the percentage of liquid lost [83]; therefore, animals with a greater amount of collagen (older animals) have a greater stability of the collagen molecule, requiring greater heat treatment to break down this molecule. A stable structure is less thermo-soluble; thus, they need higher temperatures and cooking times to reach tenderness resulting from food preparation, causing greater loss [84]. In general, beef shows a CL range between 13.1% and 34.54% [85], and the Nellore breed has meat with lower CL rates [86]. In the present study, we found average CL values from 27.01 to 31.76, which are consistent with what was expected for beef. The results showed a tendency between treatments (*p* = 0.08) and an interaction between treatments and aging times to which the steaks were submitted, indicating possible differences between the groups with longer maturation times, requiring further studies to corroborate this conclusion. The authors in [65] found no differences in CL in bulls whose mothers were fed high or low protein prior to conception and/or in the first trimester of their pregnancies. However, Ref. [87] conducted a review of studies on the effects of fetal programming and maternal nutrition and found the influence of age and gender on CL. This result was also found by [88], in which maternal malnutrition had an effect on the muscle of lambs with increased loss in the *Semitendinosus* muscle of males.

## 5. Conclusions

In this study, prenatal nutritional stimulation showed limited effects on muscle development and carcass traits in Nellore cattle. Although the effects of maternal nutrition on meat quality traits were mild, scientific evidence shows the interference of nutrient intake by mothers on the control of myogenesis and adipogenesis of offspring. In addition, the antagonism of the results found in the literature is related to the level of restriction or the supply of nutrients and the specificity of the nutrient used. Furthermore, cows mobilized their reserves to meet the demands of the fetuses, ensuring the supply of nutrients for fetal development in the treatment without supplementation.

## Figures and Tables

**Table 1:** Ingredients and nutrient content of the supplement for dams.

Ingredients (Dry Matter)	Mineral Supplement	Protein-Energy Supplement
Corn (%)	35.00	60.00
Soybean meal (%)	-	30.00
Dicalcium phosphate (%)	10.00	-
Urea 45% (%)	-	2.50
Salt (%)	30.00	5.00
Minerthal 160 MD (%) *	25.00	2.50
Nutrients	Mineral Supplement	Protein-Energy Supplement
Total digestible nutrients (%)	26.76	67.55
Crude protein (%)	2.79	24.78
Non-protein nitrogen (%)	-	7.03
Acid detergent fiber (%)	1.25	4.76
Neutral detergent fiber (%)	4.29	11.24
Fat (%)	1.26	2.61
Calcium (g/kg)	74.11	6.20
Phosphorus (g/kg)	59.38	7.24

* Mineral premix composition (Minerthal company, São Paulo, Brazil): Calcium = 8.6 g/kg; Cobalt = 6.4 mg/kg; Copper = 108 mg/kg; Sulfur = 2.4 g/kg; Fluorine = 64 mg/kg; Phosphorus = 6.4 g/kg; Iodine = 5.4 mg/kg; Manganese = 108 mg/kg; Selenium = 3.2 mg/kg; Zinc = 324 mg/kg; Sodium monensin = 160 mg/kg [23].

**Table 2:** Nutrients in pastures consumed by dams in the different groups (mean ± standard error of the mean).

Forage Nutrients	NP	PP	FP
CP% (crude protein)	7.38 ± 0.70	7.82 ± 0.93	7.40 ± 0.93
TDN% (total digestible nutrients)	63.1 ± 0.59	64.1 ± 0.95	61.4 ± 0.86
NDF% (neutral detergent fiber)	59.0 ± 1.49	61.4 ± 2.06	58.4 ± 1.67
Ca% (calcium)	0.38 ± 0.04	0.35 ± 0.02	0.39 ± 0.03
P% (phosphorus)	0.19 ± 0.01	0.19 ± 0.01	0.17 ± 0.01

NP—not programmed; PP—partially programmed; FP—full programming [23].

**Table 3:** Carcass characteristics, total lipids, and marbling score of males supplemented during pregnancy.

Traits	Treatments			
	NP	PP	FP	*p*-Value ^1^
Hot carcass weight (kg)	348.1 ± 4.61	352.7 ± 4.70	356.1 ± 4.73	0.61
Cold carcass weight (kg)	344.2 ± 6.12	349.3 ± 4.79	353.9 ± 4.81	0.58
Rib eye area (cm^2^)	97.6 ± 1.05	98.2 ± 0.96	97.4 ± 0.92	0.70
Subcutaneous fat thickness (mm)	7.81 ± 0.28	8.21 ± 0.33	8.69 ± 0.39	0.08
Total lipids (%)	1.79 ± 0.11	1.64 ± 0.08	1.69 ± 0.12	0.47
Marbling score	3.18 ± 0.36	3.17 ± 0.32	4.10 ± 0.35	0.15

NP—not programmed; PP—partially programmed; FP—full programming. ^1^—*p* value between treatments.

**Table 4:** pH and color after shelf life of 1, 3, and 5 days of programmed bulls.

Shelf life	Treatments					
	NP	PP	FP	*p*-Value ^1^	*p*-Value ^2^	*p*-Value ^3^
		pH				
D1	5.59 ± 0.03	5.57 ± 0.03	5.57 ± 0.03	0.89		
D3	5.63 ± 0.02	5.63 ± 0.02	5.62 ± 0.02	0.64	0.61	0.97
D5	5.62 ± 0.02	5.63 ± 0.02	5.62 ± 0.02	0.65		
		L*				
D1	41.76 ± 0.35	41.82 ± 0.34	42.90 ± 0.45	0.10		
D3	42.94 ± 0.39	43.12 ± 0.52	43.85 ± 0.41	0.38	0.01 *	0.81
D5	42.62 ± 0.44	41.78 ± 0.50	43.31 ± 0.65	0.26		
		a*				
D1	24.34 ± 0.30	23.39 ± 0.22	23.90 ± 0.34	0.25		
D3	22.68 ± 0.27	22.59 ± 0.29	22.65 ± 0.34	0.81	0.31	0.80
D5	20.83 ± 0.36	20.70 ± 0.30	21.02 ± 0.52	0.80		
		b*				
D1	17.23 ± 0.22	16.30 ± 0.21	16.62 ± 0.28	0.09		
D3	17.80 ± 0.21	17.62 ± 0.20	17.82 ± 0.29	0.57	0.07	0.58
D5	16.72 ± 0.25	16.51 ± 0.23	16.75 ± 0.31	0.75		

D1; D3; D5 are the days of samples displayed on the shelf. L*; a*; b* are colorimetric illuminants. NP—not programmed; PP—partially programmed; FP—full programming. ^1^—*p* value between treatments; ^2^—*p* value on repeated measures over time; ^3^—*p* value of the interaction treatment × time; *—statistical difference *p* < 0.05.

**Table 5:** pH and color after maturation of 0, 7, and 14 days of programmed bulls.

Maturation	Treatments					
	NP	PP	FP	*p*-Value ^1^	*p*-Value ^2^	*p*-Value ^3^
		pH				
D0	5.68 ± 0.07	5.58 ± 0.03	5.57 ± 0.03	0.41		
D7	5.61 ± 0.02	5.58 ± 0.02	5.59 ± 0.02	0.55	0.45	0.62
D14	5.64 ± 0.02	5.65 ± 0.02	5.64 ± 0.01	0.85		
		L*				
D0	42.30 ± 0.42	42.91 ± 0.40	42.70 ± 0.46	0.63		
D7	45.11 ± 0.37	45.00 ± 0.37	45.61 ± 0.42	0.46	0.35	0.75
D14	46.19 ± 0.42	46.00 ± 0.51	46.81 ± 0.41	0.42		
		a*				
D0	23.54 ± 0.31	24.14 ± 0.25	23.53 ± 0.32	0.29		
D7	26.11 ± 0.25	26.40 ± 0.22	25.95 ± 0.27	0.14	0.02 *	0.97
D14	26.42 ± 0.24	26.75 ± 0.23	26.21 ± 0.30	0.23		
		b*				
D0	16.57 ± 0.24	16.87 ± 0.24	16.50 ± 0.32	0.61		
D7	19.20 ± 0.25	19.35 ± 0.22	19.30 ± 0.24	0.45	0.36	0.97
D14	19.69 ± 0.15	19.87 ±0.20	19.65 ± 0.25	0.67		

D0; D7; D14 are the days the samples underwent maturation. L*; a*; b* are colorimetric illuminants. NP—not programmed; PP—partially programmed; FP—full programming. ^1^—*p* value between treatments; ^2^—*p* value on repeated measures over time; ^3^—*p* value of the interaction treatment × time; *—statistical difference *p* < 0.05.

**Table 6:** Cooking loss (%) and shear force (N) of programmed bulls’ meat.

Traits	Treatments					
	NP	PP	FP	*p*-Value ^1^	*p*-Value ^2^	*p*-Value ^3^
		CL (%)				
D0	27.67 ± 0.47	27.01 ± 0.51	28.39 ± 0.56	0.13		
D7	29.69 ± 0.43	29.74 ± 0.45	29.06 ± 0.41	0.51	0.41	0.04 *
D14	29.81 ± 0.92	31.76 ± 0.74	28.77 ± 0.70	0.08		
		WBSF (N)				
D0	79.11 ± 1.47	77.45 ± 1.81	78.75 ± 2.29	0.51		
D7	63.07 ± 1.40	65.75 ± 1.56	61.45 ± 1.81	0.16	0.31	0.30
D14	56.29 ± 2.05	59.05 ± 1.84	53.94 ± 1.87	0.21		

CL—cooking loss; WBSF—Warner–Bratzler shear force. D0; D7; D14 are the days that the samples underwent maturation. NP—not programmed; PP—partially programmed; FP—full programming ^1^—*p* value between treatments; ^2^—*p* value on repeated measures over time; ^3^—*p* value of the interaction treatment x time; *—statistical difference *p* < 0.05.

## Data Availability

None of the data were deposited in an official repository. The raw data supporting the conclusion of this article will be made available by the authors, without undue reservation.
